# Molecular detection and *VP1*-based phylogenetic characterization of Foot-and-mouth disease virus serotype O circulating in cattle in Aceh, Indonesia

**DOI:** 10.14202/vetworld.2026.2221-2232

**Published:** 2026-05-27

**Authors:** M. Daud AK, Mahdi Abrar, Teuku Reza Ferasyi, Darniati Darniati, Erina Erina, Tongku Nizwan Siregar, Surachmi Setiyaningsih

**Affiliations:** 1 Department of Microbiology, Faculty of Veterinary Medicine, Universitas Syiah Kuala, Banda Aceh, Indonesia; 2 Department of Veterinary Public Health, Faculty of Veterinary Medicine, Universitas Syiah Kuala, Banda Aceh, Indonesia; 3 Centre for Tropical Veterinary Studies-One Health Collaboration Centre, Universitas Syiah Kuala, Banda Aceh, Indonesia; 4 Laboratory of Reproduction, Faculty of Veterinary Medicine, Universitas Syiah Kuala, Banda Aceh, Indonesia; 5 Department of Animal Infectious Diseases and Veterinary Public Health, Faculty of Veterinary Medicine, IPB University, Bogor, Indonesia

**Keywords:** Aceh, cattle, foot-and-mouth disease virus, Ind-2001e, molecular epidemiology, phylogenetic analysis, serotype O, *VP1* gene

## Abstract

**Background and Aim:**

Foot-and-mouth disease (FMD) remains a major transboundary animal disease affecting livestock production and trade worldwide. Following the re-emergence of FMD virus (FMDV) in Indonesia in 2022, repeated outbreaks have continued to occur in several provinces, including Aceh. However, molecular information regarding circulating strains in Aceh remains limited. This study aimed to detect FMDV in clinically affected cattle in Aceh, Indonesia, using serological and molecular approaches and to characterize the phylogenetic relationship of the detected virus based on the *VP1* gene.

**Materials and Methods:**

Serum and swab samples were collected from clinically suspected cattle in Aceh Besar District, Aceh Province, Indonesia, during 2024. Antibodies against FMDV non-structural proteins were detected using non-structural protein enzyme-linked immunosorbent assay. Viral RNA was extracted from nasopharyngeal and oral swabs and examined using reverse transcription polymerase chain reaction targeting the 5′ untranslated region and 3D gene. Positive samples were further analyzed using serotype O-specific *VP1* primers. Amplicons were sequenced using the Sanger method, and phylogenetic analysis was performed using the Neighbor-Joining method with the Kimura 2-parameter model and 1,000 bootstrap replicates in MEGA version 12.

**Results:**

Non-structural protein antibody detection showed positive or weak-positive reactions in a subset of clinically affected cattle, while all clinically uninfected cattle were negative. Reverse transcription polymerase chain reaction confirmed FMDV RNA in 70% of tested swab samples, producing the expected 328 bp and 644 bp amplicons. *VP1* amplification generated an approximately 1,135 bp fragment, with 56°C identified as the optimal annealing temperature. Phylogenetic analysis demonstrated that the representative isolate, O/AcehBesar/ 01A/2024, clustered within the Middle East-South Asia topotype, Ind-2001e sub-lineage, together with Indonesian outbreak strains reported during 2022. The isolate shared 96% nucleotide identity with Indonesian serotype O isolates and showed close genetic relationships with strains from several Asian and trans-regional countries.

**Conclusion:**

The study confirmed the continued circulation of FMDV serotype O belonging to the Middle East-South Asia/Ind-2001e sub-lineage in Aceh, Indonesia. The findings provide updated *VP1*-based molecular evidence from Aceh and emphasize the importance of integrating serological and molecular surveillance for outbreak investigation, lineage monitoring, and future vaccine-matching studies.

## INTRODUCTION

Foot-and-mouth disease (FMD) remains one of the most important transboundary animal diseases affecting cloven-hoofed animals worldwide [1, 2]. The disease causes major economic losses through reduced productivity, restrictions on animal movement, and disruption of domestic and international trade [3, 4]. FMD virus (FMDV), a member of the genus Aphthovirus in the family Picornaviridae, comprises seven immunologically distinct serotypes with substantial genetic and antigenic diversity, which complicates disease control and vaccine selection [5]. In endemic and re-emerging settings, rapid and accurate diagnosis is essential for outbreak response. Reverse transcription polymerase chain reaction (RT-PCR) has substantially improved the detection of FMDV in clinical samples, while serological assays such as non-structural protein (NSP) enzyme-linked immunosorbent assay (ELISA) support surveillance by distinguishing infected animals from vaccinated animals [6, 7]. In addition, sequence-based characterization, particularly targeting the *VP1* gene, is widely used for serotype identification, topotype classification, and molecular tracing of outbreak strains [8].

Indonesia regained FMD-free status in 1990; however, the disease re-emerged in April 2022 and subsequently spread rapidly across multiple provinces [9]. Aceh was among the affected regions and has remained epidemiologically important because of repeated outbreaks and continued cattle movement within the province [10]. Previous reports from Indonesia have primarily focused on outbreak occurrence, rapid diagnostic testing, and national-scale epidemiological descriptions, whereas detailed molecular characterization data from Aceh remain scarce [11]. The limited availability of localized *VP1*-based phylogenetic information restricts understanding of lineage persistence, viral evolution, and regional transmission dynamics within Aceh and neighboring provinces [12]. Furthermore, the relationship between currently circulating Aceh isolates and previously reported Indonesian outbreak strains has not been comprehensively evaluated using combined serological and molecular approaches. This limitation reduces the capacity to support evidence-based surveillance, outbreak tracing, and future vaccine-matching strategies.

Recent molecular epidemiological studies have demonstrated that *VP1* gene analysis remains a robust approach for investigating FMDV transmission patterns, genetic relationships, and lineage distribution across endemic and re-emerging regions [13, 14]. However, despite recurrent outbreaks in Aceh following the 2022 national re-emergence, there is still insufficient updated molecular evidence regarding the circulating serotype O strains in this province. In addition, few studies have integrated NSP antibody detection with RT-PCR and *VP1* sequencing to evaluate both serological exposure and active viral circulation in cattle populations from Aceh. Therefore, a comprehensive investigation combining serological and molecular approaches is needed to strengthen local outbreak investigations and improve regional molecular surveillance.

This study aimed to investigate FMDV in cattle from Aceh, Indonesia, using serological and molecular approaches and to characterize the phylogenetic relationship of the detected virus based on the *VP1* gene. Specifically, the objectives were: (1) to detect FMDV infection using NSP antibody ELISA and RT-PCR; (2) to identify the serotype of the detected virus through *VP1* gene amplification; and (3) to assess the genetic relationship of the Aceh isolate with Indonesian and international reference strains using phylogenetic analysis. We hypothesized that serotype O viruses genetically related to previously reported Indonesian outbreak strains continue to circulate in Aceh. The findings of this study provide updated localized molecular evidence from Aceh and contribute to ongoing FMDV surveillance, lineage monitoring, and future control planning in Indonesia.

## MATERIALS AND METHODS

### Ethical approval

All animal handling, restraint, and sample collection procedures were conducted in accordance with national veterinary ethical guidelines and institutional biosafety regulations for infectious disease investigations in livestock. The study protocol was reviewed and approved by the Institutional Animal Ethics Committee, Faculty of Veterinary Medicine, Universitas Syiah Kuala, Banda Aceh, Indonesia (Approval No. 291/KEPH/I/2024). Animal welfare considerations were strictly maintained during field sampling to minimize stress, discomfort, and unnecessary handling of cattle. Sampling procedures were performed by trained veterinary personnel using appropriate restraint techniques and personal protective equipment. No experimental infection or live virus propagation was conducted during this study.

### Study period and location

**Figure 1:**
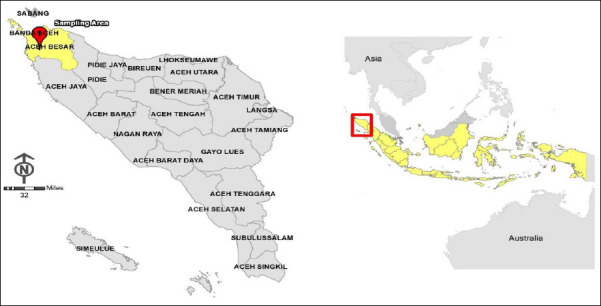
Study area of sample collection located in Aceh Besar District (yellow), Aceh Province, Indonesia.

The study was conducted from January to December 2024 in Aceh Besar District, Aceh Province, Indonesia. Field samples were collected from cattle raised in Darul Kamal (approximately 5.500832° N, 95.3301567° E) and Suka Makmur (approximately 5.488542° N, 95.379858° E) sub-districts (Figure 1). These areas were selected because they had reported cattle with clinical signs consistent with FMD and remained epidemiologically relevant for outbreak surveillance following the national re-emergence of FMD in Indonesia in 2022. Aceh Besar continues to experience repeated FMD outbreaks and active cattle movement, making the region important for molecular epidemiological investigation.

### Study design

This study used an observational laboratory-based molecular epidemiology design to detect and characterize FMDV associated with cattle outbreaks in Aceh, Indonesia. The investigation combined serological screening, molecular detection, serotype confirmation, *VP1* gene amplification, sequencing, and phylogenetic analysis of field samples collected from clinically suspected cattle. The study focused on evaluating the presence of circulating FMDV serotype O strains and determining their phylogenetic relationship with Indonesian and international reference isolates.

### Sample collection and handling

**Clinical swab collection:** Nasopharyngeal and oral swabs were collected from cattle showing clinical signs suggestive of FMD, including hypersalivation, vesicular lesions in the oral cavity, lesions on the feet, and lameness. Animals vaccinated within the previous 6 months, calves younger than 6 months, and animals with unrelated systemic illness were excluded from the study. All animals were physically restrained with the assistance of trained farm personnel, and sampling procedures were performed using personal protective equipment, including nitrile gloves and masks.

Swabs were placed into viral transport medium (Biocomma, Shenzhen, China), transported at 2–8°C under monitored cold-chain conditions, and stored at −80°C within 6 h after collection in an MDF-U52VAT ultra-low temperature freezer (SANYO, Temecula, CA, USA).

**Blood collection for serum:** For serological analysis, approximately 10 mL of blood was aseptically collected from the jugular vein into sterile Vacutainer tubes (Becton Dickinson, Singapore). Blood samples were allowed to clot at room temperature for 15–30 min and were subsequently centrifuged at 1,500 × g for 10 min. The resulting serum was aliquoted and stored at −80°C until further analysis.

### Serological detection of FMDV antibodies

ELISA principle and kit details: Serum samples were tested for antibodies against FMDV NSPs, particularly 3ABC, using the PrioCHECK FMDV NS Ab ELISA kit (Prionics AG, Schlieren, Switzerland). Detection of NSP antibodies was performed to support differentiation between naturally infected and vaccinated animals. The assay was conducted according to the manufacturer’s instructions, and absorbance was measured at 450 nm using a microplate reader (Bio-Rad Laboratories, Hercules, CA, USA).

### Cutoff criteria and interpretation: Percent inhibition (PI) was calculated using the following formula:

Samples with PI values ≥50% were interpreted as positive, whereas samples with PI values ≥70% were categorized as strong-positive according to the manufacturer’s recommendations. Negative, weak-positive, and strong-positive controls supplied with the kit were included in each assay run for quality assurance.

### Molecular detection of FMDV RNA

**RNA extraction and quality assessment:** Total viral RNA was extracted from swab samples using the Viral Nucleic Acid Extraction Kit (Geneaid Biotech, Taipei, Taiwan) according to the manufacturer’s instructions. RNA concentration and purity were evaluated using a NanoDrop spectrophotometer, with OD260/280 values ranging from 1.8 to 2.0. RNA integrity was additionally assessed using 1% agarose gel electrophoresis.

**RT-PCR assay conditions:** RT-PCR was performed in a total reaction volume of 25 µL using the Transcriptor One-Step RT-PCR Kit (Roche Diagnostics, Mannheim, Germany). Two primer pairs targeting the 5′ untranslated region (5′ UTR; expected amplicon size 328 bp) and the 3D gene (expected amplicon size 644 bp) were used to improve detection reliability (Table 1). The primer sequences were 1F (5′-GCCTGGTCTTTCCAGGTCT-3′) and 1R (5′-CCAGTCCCC TTCTCAGATC-3′), and FM8 (5′-GTCAGACCTTCCTGAAGGACG-3′) and FM9 (5′-CCTTTGTCGCTTTT GTCAGCTGG-3′) [16, 17]. A no-template negative control was included in each run to monitor contamination.

**Table 1:** Primer sets used for FMD virus detection by reverse transcription polymerase chain reaction.

Primer	Sequence (5′–3′)	Target	Amplicon size	Reference
1F	GCCTGGTCTTTCCAGGTCT	5′ UTR	328 bp	[16]
1R	CCAGTCCCCTTCTCAGATC			
FM8	GTCAGACCTTCCTGAAGGACG	3D	644 bp	[17]
FM9	CCTTTGTCGCTTTTGTCAGCTGG			

### Agarose gel electrophoresis

Amplified products were separated on 1% agarose gel using GeneRuler 1 kb DNA Ladder (Thermo Fisher Scientific, Waltham, MA, USA), stained with ethidium bromide, and visualized under ultraviolet illumination using a UV transilluminator (Cole-Parmer, Chicago, USA). Bands corresponding to the expected amplicon size were interpreted as positive.

### *VP1* gene amplification and serotype identification

The *VP1* gene was amplified from selected RT-PCR-positive samples using serotype O-specific primers targeting an approximately 1,135 bp region. The primers used were O-C272F (5′-TBGCRGGNCTYGCCCAGTACTAC-3′), O-1C605xF (5′-TGGCTAGTGCTGGTAAAGACTTTGAG-3′), and EUR-2B52R (5′-GACATGTCCTCCTGCATCTGGTT GAT-3′). These primers have been widely applied for molecular characterization of FMDV serotype O [15].

Amplification was performed using the following thermal profile: initial denaturation at 94°C for 7 min, followed by 30 cycles of denaturation at 94°C for 15 s, annealing at 56°C for 30 s, and extension at 68°C for 1 min. Reactions were conducted using a Bio-Rad MJ Mini Thermal Cycler (Bio-Rad Laboratories, Hercules, CA, USA). Annealing temperature optimization identified 56°C as the optimal condition, producing clear and specific amplification bands. The *VP1* gene was selected because it contains major antigenic determinants and is widely used for serotype differentiation and phylogenetic classification.

### Sequencing and data processing

**Sanger sequencing workflow:** Purified VP1 amplicons were subjected to bidirectional Sanger sequencing at Macrogen (Seoul, South Korea) using BigDye Terminator v3.1 chemistry and an ABI 3730XL automated sequencer.

**Sequence editing and alignment:** Sequence chromatograms were manually inspected, and low-quality terminal regions were trimmed before consensus sequence assembly. Preliminary sequence identity was evaluated using BLASTn. Multiple sequence alignment was performed using MUSCLE implemented in MEGA version 12 [18]. The representative VP1 sequence generated in this study was submitted to GenBank (accession number PZ243349).

### Phylogenetic analysis

**Tree construction:** Phylogenetic analysis was conducted using the Neighbor-Joining method with the Kimura 2-parameter substitution model implemented in MEGA version 12 [19]. Branch support was evaluated using 1,000 bootstrap replicates.

**Reference sequence selection:** Reference VP1 sequences representing Indonesian outbreak strains and selected international FMDV serotype O strains, particularly members of the Middle East-South Asia (ME-SA) topotype and Ind-2001e sub-lineage, were retrieved from GenBank for comparative analysis. These reference sequences were included to position the Aceh isolate within a broader molecular epidemiological framework. A complete list of reference sequences used for phylogenetic analysis, including accession number, country, year, and lineage designation, is provided in Supplementary Table S1.

**Biosafety and containment measures:** All procedures involving potentially infectious materials were conducted using appropriate biosafety precautions. Viral inactivation occurred during RNA extraction, and no live virus propagation was performed. Laboratory personnel followed institutional biosafety and containment procedures to minimize the risk of exposure and environmental contamination.

## RESULTS

### Detection of anti-NSP antibodies against FMDV

Detection of antibodies against FMDV NSPs is important for distinguishing naturally infected animals from vaccinated animals because NSPs are produced during active viral replication and are generally absent from purified vaccines. This principle has been recognized by World Organization for Animal Health, which recommends NSP-based serological assays for disease surveillance and post-vaccination monitoring [20].

In this study, NSP antibody detection was performed using the PrioCHECK FMDV NS Ab ELISA kit on cattle sera collected in Aceh, Indonesia. The assay fulfilled the manufacturer’s validation criteria. The average optical density (OD450) values of the negative controls were above 1.00, whereas the weak-positive and strong-positive controls showed PI values above the required thresholds. The negative control wells showed OD450 values of 2.191 and 2.182, whereas the weak-positive and strong-positive controls showed PI values ranging from 66%–67% and 88%–89%, respectively [21]. These findings confirmed that the assay performed adequately under the conditions of the study.

A total of 24 serum samples were analyzed, including 18 samples collected from clinically infected cattle and six samples collected from cattle classified as uninfected based on clinical and epidemiological assessment (Table 2). Among the clinically infected cattle, five samples (27.8%) were classified as weak-positive (PI ≥50% and <70%), whereas one sample (5.6%) was classified as positive (PI ≥70%). The remaining 12 samples (66.7%) were negative. All six samples from the uninfected group were negative for anti-NSP antibodies.

**Table 2:** Detection of antibodies against NSPs of FMDV in infected and uninfected cattle sera collected after reported FMD outbreaks in Aceh, Indonesia.

Sample	OD450 tested sample	PI (%)	Result interpretation	Description
Negative	2.19	0	Negative	Negative control
Negative	2.18	0	Negative	Negative control
Weak-positive	0.71	67	Weak-positive	Weak-positive control
Weak-positive	0.74	66	Weak-positive	Weak-positive control
Positive	0.24	89	Positive	Positive control
Positive	0.27	88	Positive	Positive control
AcehBesar/2024/1	0.99	55	Weak-positive	Infected cattle
AcehBesar/2024/2	1.70	22	Negative	Infected cattle
AcehBesar/2024/3	1.91	12	Negative	Infected cattle
AcehBesar/2024/4	1.06	51	Weak-positive	Infected cattle
AcehBesar/2024/5	1.82	17	Negative	Infected cattle
AcehBesar/2024/6	2.07	6	Negative	Infected cattle
AcehBesar/2024/7	1.94	12	Negative	Infected cattle
AcehBesar/2024/8	0.85	61	Weak-positive	Infected cattle
AcehBesar/2024/9	0.90	59	Weak-positive	Infected cattle
AcehBesar/2024/10	1.58	28	Negative	Uninfected cattle
AcehBesar/2024/11	0.82	62	Weak-positive	Infected cattle
AcehBesar/2024/12	0.55	75	Positive	Infected cattle
BandaAceh/2024/1	1.85	15	Negative	Uninfected cattle
BandaAceh/2024/2	2.05	6	Negative	Uninfected cattle
BandaAceh/2024/3	1.70	22	Negative	Uninfected cattle
BandaAceh/2024/4	1.66	24	Negative	Uninfected cattle
BandaAceh/2024/5	1.52	31	Negative	Uninfected cattle
BandaAceh/2024/6	1.80	18	Negative	Uninfected cattle

The ELISA results demonstrated variation in PI values among clinically infected cattle. The strongest antibody response was observed in sample AcehBesar/2024/12, which showed a PI value of 75% and was classified as positive. Samples AcehBesar/2024/1, AcehBesar/2024/4, AcehBesar/2024/8, AcehBesar/2024/9, and AcehBesar/ 2024/11 were classified as weak-positive, indicating detectable but relatively lower anti-NSP antibody responses. In contrast, all samples from the uninfected group remained below the cutoff value.

The overall serological profile suggested that only a subset of clinically affected cattle had detectable anti-NSP antibodies at the time of sampling. This pattern may reflect differences in infection stage, timing of sample collection relative to seroconversion, variation in viral replication, or differences in host immune responses [22]. The absence of anti-NSP antibodies in the uninfected group supported the field classification of these animals and indicated good assay specificity under the conditions of this study [23].

These findings further emphasized the importance of combining serological and molecular approaches during outbreak investigations. NSP-ELISA is useful for identifying prior or ongoing infection; however, antibody responses may not yet be detectable during the early phase of infection [24]. Therefore, serological testing alone may underestimate active infection during outbreak situations.

### RT-PCR detection of FMDV RNA

RT-PCR was used to detect FMDV RNA in nasopharyngeal and oral swab samples collected from cattle in Aceh, Indonesia. Two primer sets were used: 1F/1R targeting the 5′ UTR to amplify a 328 bp fragment and FM8/9 targeting the 3D gene to amplify a 644 bp fragment. These primer pairs have been widely used and validated for FMDV detection and serotype identification [25, 26].

**Figure 2:**
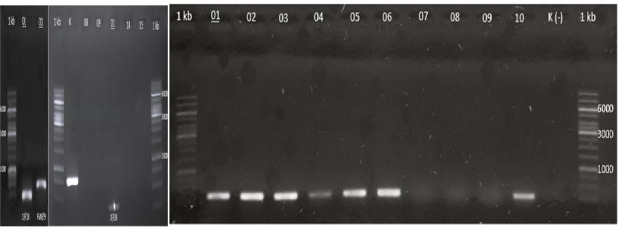
Electrophoresis results using primer pair 1F/1R targeting the 5′ UTR region (328 bp amplicon) and primer pair FM8/9 targeting the 3D region (644 bp amplicon) on nasopharyngeal and oral swab samples (sample code 01). Electrophoresis was performed at 100 V for 30 min and showed clear positive bands corresponding to FMDV without visible nonspecific amplification or contamination.

Gel electrophoresis showed positive amplification in seven of 10 tested swab samples (Figure 2). The expected amplicons of 328 bp and 644 bp were clearly observed in the positive samples, whereas no amplification was detected in the negative control, indicating the absence of contamination during the assay.

The use of two genomic targets increased diagnostic confidence by combining detection of conserved genomic regions with an additional confirmatory target. The clear amplification pattern and absence of nonspecific bands indicated that the assay performed reliably on the tested field samples. Detection of FMDV RNA in 70% of the tested swab samples supported ongoing viral circulation in the outbreak area and confirmed the usefulness of RT-PCR for field-based FMDV detection in Aceh [27].

### *VP1* gene amplification and serotype characterization

**Figure 3:**
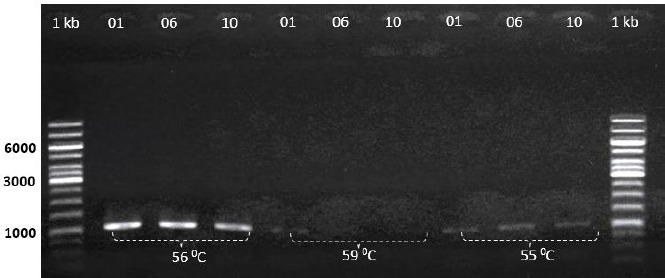
RT-PCR amplification of the *VP1* gene fragment (1,135 bp) from three isolates (sample codes: 01, 06, and 10) using three annealing temperature conditions. The 56°C condition produced the clearest and brightest amplification bands. Electrophoresis was performed at 100 V for 30 min and demonstrated amplification at the expected product size of 1,135 bp.

*VP1* gene amplification targeting an approximately 1,135 bp fragment was performed on three FMDV-positive samples: AcehBesar/2024/01, AcehBesar/2024/06, and AcehBesar/2024/10. Amplification was evaluated using three annealing temperatures, namely 55°C, 56°C, and 59°C (Figure 3). Among these conditions, 56°C produced the clearest and most specific amplification bands at the expected product size, whereas amplification at 55°C and 59°C appeared weaker and less distinct.

The *VP1* gene encodes a major capsid protein and is widely used for serotype identification and molecular epidemiological analysis of FMDV [28]. Successful amplification using serotype O-specific primers supported the identification of serotype O in the tested samples. This finding was consistent with the RT-PCR detection results and confirmed the presence of serotype O in outbreak samples collected from Aceh.

The *VP1* amplification step provided the basis for downstream sequencing and phylogenetic analysis. Optimization of the annealing temperature identified 56°C as the optimal condition because it yielded reproducible and specific amplification products [29]. Among the three *VP1*-positive amplicons, only one generated sequence data of sufficient quality for consensus sequence assembly and subsequent phylogenetic analysis.

### Phylogenetic analysis of the VP1 gene of the Aceh FMDV isolate

**Figure 4:**
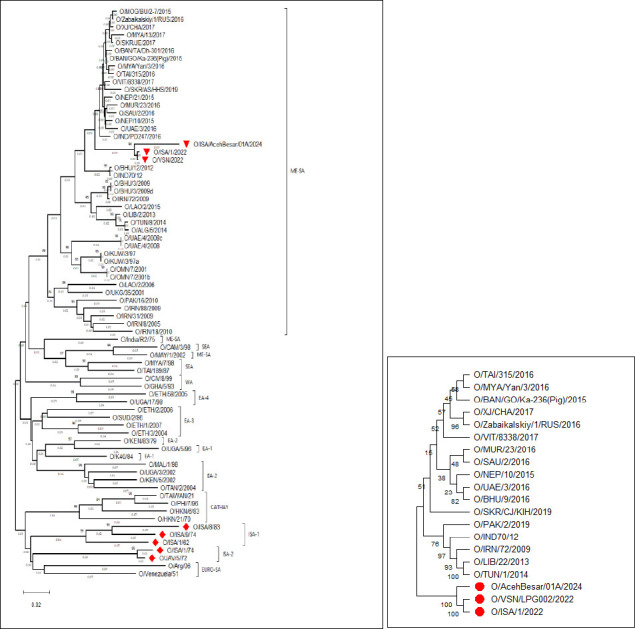
Phylogenetic analysis of the VP1 gene of FMDV serotype O isolates. A Neighbor-Joining tree was constructed based on VP1 nucleotide sequences (1,135 bp) using MEGA version 12 with 1,000 bootstrap replicates. The Aceh isolate, O/AcehBesar/01A/2024, clustered within the ME-SA topotype, Ind-2001e sub-lineage, together with Indonesian isolates from the 2022 outbreak (O/ISA/1/2022 and O/VSN/LPG002/2022), showing 96% nucleotide identity and strong bootstrap support (100%). The tree also included representative Ind-2001-related strains from Pakistan, India, Iran, Tunisia, the United Arab Emirates, Saudi Arabia, Mauritius, Nepal, and Libya.

Phylogenetic reconstruction based on the *VP1* gene demonstrated that the Aceh isolate, O/AcehBesar/ 01A/2024, clustered within the ME-SA topotype, specifically within the Ind-2001e sub-lineage. In the phylogenetic tree, the Aceh isolate grouped closely with Indonesian isolates reported during the 2022 outbreak, including O/ISA/1/2022 and O/VSN/LPG002/2022, showing 96% nucleotide identity and 100% bootstrap support. These isolates formed a well-supported monophyletic cluster, indicating a close genetic relationship. In contrast, the Aceh isolate was clearly separated from older Indonesian strains such as O/ISA/8/83 and O/ISA/1/74, which belong to the ISA topotype (Figure 4).

The Aceh isolate also demonstrated phylogenetic relatedness to other serotype O Ind-2001 lineage strains reported from Pakistan, India, Iran, Tunisia, Libya, the United Arab Emirates, Nepal, Saudi Arabia, and Mauritius. This broader clustering pattern was consistent with the previously reported circulation of the Ind-2001 lineage across multiple geographic regions.

These findings indicated that the Aceh 2024 isolate belongs to the same broad lineage that has circulated in Indonesia since the 2022 outbreak. The phylogenetic placement supports the continued detection of the Ind-2001e sub-lineage in Aceh and provides updated molecular epidemiological evidence from this province.

## DISCUSSION

### Phylogenetic relationship and lineage continuity of the Aceh isolate

The phylogenetic placement of the Aceh isolate within the ME-SA/Ind-2001e sub-lineage was consistent with previous reports demonstrating widespread circulation of this lineage across South Asia, Southeast Asia, the Middle East, and parts of Africa [30–32]. The close clustering of O/AcehBesar/01A/2024 with Indonesian outbreak isolates reported during 2022 indicated that the Aceh virus is genetically related to strains associated with the recent re-emergence of FMD in Indonesia [33]. Based on the present findings, this relationship is more consistent with continued circulation or recurrence of a closely related viral population rather than introduction of a clearly distinct lineage.

The relatively high nucleotide similarity observed between the Aceh isolate and Indonesian isolates from the 2022 outbreak suggested limited divergence among the compared *VP1* sequences [34]. This pattern may indicate persistence of a genetically related lineage within Indonesia following the national outbreak. However, because the present study included a limited number of samples and only one representative sequence subjected to phylogenetic analysis, the findings should be interpreted cautiously. Although the results support lineage continuity at the provincial level, they do not independently establish the complete transmission history or origin of the circulating virus.

### Utility of *VP1* gene analysis in molecular epidemiology

Use of the *VP1* gene in the present study provided adequate resolution for lineage assignment and comparison with previously published reference strains [35]. Full-length or near-full-length *VP1* sequence analysis has been widely used in FMDV molecular epidemiology because it supports serotype confirmation and enables comparison among genetically related outbreak strains. In the present investigation, *VP1*-based analysis confirmed that the Aceh isolate belonged to serotype O and clustered within the Ind-2001e sub-lineage together with recent Indonesian isolates.

The observed phylogenetic relationship between the Aceh isolate and other regional strains also supports the usefulness of *VP1*-based characterization for tracing lineage distribution and monitoring outbreak dynamics. Molecular surveillance based on *VP1* sequence data remains particularly valuable in endemic and re-emerging settings because it allows rapid comparison of newly detected isolates with regional and international reference strains.

### Interpretation of combined serological and molecular findings

The combined serological and molecular findings also helped explain the outbreak status of the sampled cattle population. Anti-NSP antibodies were detected only in a subset of clinically affected cattle, whereas viral RNA was detected in several swab samples. This pattern is biologically plausible during field outbreaks because animals may be sampled at different stages of infection. Some animals may have been sampled before seroconversion, whereas others may have developed detectable antibodies after active viral replication had already occurred [36].

These findings support the importance of integrating serological and molecular testing during outbreak investigations rather than relying on a single diagnostic method [37]. RT-PCR is highly effective for detecting active infection during the acute phase, whereas NSP antibody detection provides evidence of exposure and ongoing immune response. The combined application of these approaches therefore improves outbreak confirmation and epidemiological interpretation in field conditions.

### Implications for surveillance and disease control

From a disease control perspective, confirmation of serotype O and assignment of the Aceh isolate to the Ind-2001e lineage are relevant for molecular surveillance programs in Indonesia [38]. The findings support continued monitoring of circulating field strains, particularly in provinces such as Aceh where repeated outbreaks have been reported since the 2022 national re-emergence of FMD.

At the same time, the present study did not include antigenic characterization, virus neutralization testing, or vaccine-matching assays. Therefore, implications regarding vaccine performance should be interpreted cautiously. The current findings support the need for continued molecular surveillance and future vaccine-matching investigations; however, the available data do not independently demonstrate vaccine mismatch or reduced vaccine efficacy [39].

### Study limitations and future perspectives

The number of serum and swab samples was limited, only a small number of *VP1*-positive amplicons was obtained, and phylogenetic interpretation was based on a single representative sequence. In addition, no full-genome sequencing or antigenic characterization was performed. These limitations reduce the strength of epidemiological inference and prevent broader conclusions regarding viral spread, persistence mechanisms, and vaccine effectiveness from being made with certainty.

Future studies should include larger sample sizes, expanded geographic sampling, full-genome sequencing, and antigenic characterization to better understand FMDV transmission dynamics in Aceh and other Indonesian provinces. Integration of molecular epidemiology with animal movement data and vaccination history would also strengthen outbreak tracing and surveillance strategies.

Overall, the present study adds localized molecular evidence from Aceh to the growing body of data regarding FMDV serotype O circulation in Indonesia. The clustering of the Aceh isolate within the Ind-2001e sub-lineage supports continued detection of this lineage in a high-risk province and highlights the importance of sustained genomic surveillance to support outbreak tracing, lineage monitoring, and future disease control planning [40].

## CONCLUSION

This study confirmed the detection and continued circulation of FMDV serotype O in cattle from Aceh, Indonesia, using integrated serological and molecular approaches. RT-PCR successfully detected FMDV RNA in 70% of tested swab samples, whereas NSP antibody ELISA identified anti-NSP antibodies in a subset of clinically affected cattle, demonstrating variation in immune response according to the stage of infection. *VP1* gene amplification generated the expected 1,135 bp fragment under optimized assay conditions, and phylogenetic analysis demonstrated that the representative Aceh isolate, O/AcehBesar/01A/2024, clustered within the ME-SA/Ind-2001e sub-lineage together with Indonesian outbreak isolates reported during 2022. The observed 96% nucleotide identity and strong bootstrap support indicated a close genetic relationship between the Aceh isolate and previously circulating Indonesian serotype O strains.

The findings provide updated *VP1*-based molecular evidence from Aceh, a province that has experienced repeated FMD outbreaks since the national re-emergence of the disease in 2022. The study highlights the practical value of combining serological testing with RT-PCR and phylogenetic analysis for outbreak confirmation, lineage identification, and molecular surveillance. This integrated diagnostic approach can strengthen regional outbreak investigations and support evidence-based monitoring of circulating FMDV strains in Indonesia.

A major strength of this study was the combined application of serological and molecular methods together with *VP1*-based phylogenetic characterization, allowing both detection of infection and assessment of viral genetic relationships. In addition, the inclusion of regional and international reference strains enabled placement of the Aceh isolate within a broader molecular epidemiological framework.

Nevertheless, the study had several limitations, including limited sample size, a small number of *VP1*-positive amplicons suitable for sequencing, and the absence of full-genome sequencing and antigenic characterization. Therefore, broader conclusions regarding transmission pathways, viral evolution, and vaccine effectiveness should be interpreted cautiously.

Overall, the present study contributes important localized molecular epidemiological data regarding FMDV serotype O circulation in Aceh and supports the continued detection of the Ind-2001e sub-lineage in Indonesia. Sustained genomic surveillance, expanded molecular characterization, and future vaccine-matching studies are needed to strengthen outbreak preparedness, improve lineage monitoring, and support long-term FMD control strategies in Indonesia.

## DATA AVAILABILITY

The data generated during the study are included in the manuscript.

## AUTHORS’ CONTRIBUTIONS

MD, SS and MA: Conceptualization, investigation, methodology, supervised and drafted the manuscript. MD and DN: Collected the sample and performed data analysis. MD, SS, EE: Extracted RNA, primer design, and gene amplification. TRF and TNS: Formal analysis and software validation. All authors have read and approved the final version of the manuscript.
